# Genome-wide identification and expression analysis of Phenylalanine Ammonia-Lyase (*PAL*) in cucumber

**DOI:** 10.1371/journal.pone.0334923

**Published:** 2025-11-06

**Authors:** Jiaojiao Zhang, Kunyang Wang, Shuai Meng, Qiushuang Han, Ming Gao

**Affiliations:** 1 Hebei Key Laboratory of Horticultural Germplasm Excavation and Innovative Utilization, College of Horticulture Science & Technology, Hebei Normal University of Science & Technology, Qinhuangdao, Hebei, China; 2 Analysis and Testing Center, Hebei Normal University of Science & Technology, Qinhuangdao, Hebei, China; 3 College of Horticulture, China Agricultural University, Beijing, China; Prince of Songkla University, THAILAND

## Abstract

Phenylalanine ammonia-lyase (PAL) is the key catalytic enzyme that initiates the phenylpropanoid metabolic pathway. This study identified 13 members of the cucumber *PAL* family to elucidate their characteristics and their potential role in the development of cucumber fruit astringency. All identified members exhibited high conservation and contained three conserved domains: the MIO domain, the core domain, and the insertion shielding domain. Evolutionary pressure selection analysis suggested that purifying selection was the primary driving force behind the evolution of *PAL* family members. *Cis*-acting element analysis demonstrated that *CsaPALs* responded to light, hormones, and stress. Collinearity analysis revealed collinear relationships between *AtPAL2* and *CsaPAL2*, as well as between *AtPAL4* and *CsaPAL8*. Transcriptome sequencing exhibited significant differences in the expression levels of *CsaPALs* between high- and low-astringency cucumber fruits. Furthermore, qRT-PCR analysis revealed that *CsaPAL1*, *CsaPAL2*, *CsaPAL3*, *CsaPAL9*, *CsaPAL12*, and *CsaPAL13* were significantly differentially expressed between the high- and low-astringency fruits, indicating that these genes might serve as candidates for regulating astringency in cucumber. The expression levels of these six genes were also significantly correlated with cucumber fruit tannin content. Overall, these findings provide a solid foundation for further studies on the biological roles of *PALs* in cucumber.

## Introduction

In recent years, rising living standards and improved quality of life have driven greater demand for high-quality horticultural products with taste emerging as a key priority [1]. Among various quality-related concerns, astringency in fruits and vegetables has garnered particular attention. Astringency is perceived through sensations such as dryness, roughness, contraction, and lingering adhesion in the oral cavity [2]. The main compounds responsible for this characteristic include polyphenols, organic acids, inorganic acids, and certain proteins [3–7]. In plant-based foods, astringency primarily results from polyphenolic compounds, including flavonoids, phenolic acids, and tannins [8–10]. Several studies have established tannins as the primary contributors to astringency in most horticultural crops [4,10,11]. For instance, grapes contain substantially higher levels of tannins in their pericarp, seeds, and pedicels compared to other tissues, which makes them particularly astringent [12,13]. In tea, the principal compounds responsible for astringency are tea polyphenols and flavonoid glycosides [12,14]. Similarly, the progressive accumulation of proanthocyanidins in persimmons leads to a significant increase in the astringency of persimmon fruits [15,16]. Likewise, in walnuts, the gradual increase in the content of hydrolysable tannins in the walnut seed coat during maturation significantly enhances their astringency [17].

Phenylalanine Ammonia-Lyase (PAL) functions as the initial enzyme in the phenylpropanoid metabolic pathway [18–20]. It catalyzes the conversion of phenylalanine into trans-cinnamic acid [21], which is then hydroxylated by Cinnamate-4-hydroxylase (C4H) to form p-Coumaric acid. This p-Coumaric acid is subsequently activated by 4-Coumaroyl-CoA ligase (4CL) to produce p-Coumaroyl-CoA [22]. In the flavonoid biosynthesis pathway, chalcone synthase (CHS) catalyzes the reaction between p-Coumaroyl-CoA and malonyl-CoA forming chalcone, the key precursor that initiates flavonoid production. Chalcone is further isomerized by chalcone isomerase (CHI) and hydroxylated by flavanone 3-hydroxylase (F3H) to form dihydroflavonol. Dihydroflavonol serves as the precursor for anthocyanins, tannins, and other flavonoids. Through the action of dihydroflavonol 4-reductase (DFR), dihydroflavonol is reduced to leucoanthocyanidin, which is subsequently transformed into proanthocyanidins in plants [23–25]. This metabolic pathway highlights the crucial role of *PAL* genes in driving the biosynthesis of anthocyanins, tannins, and various flavonoids in plants [26]. *PAL* was initially discovered in barley [27], and later research has identified *PAL* genes across a wide range of plant species, including Arabidopsis [28], banana [29], rice [30], wheat [31], walnut [32], and grape [33]. PAL enzymes are highly conserved and typically feature an MIO domain, which houses the enzyme’s active site. In most plant PAL enzymes, the MIO domain contains a conserved active site sequence ‘GTITASGDLVPLSYIAG’, with the characteristic Ala-Ser-Gly (ASG) motif as a key component localized within this sequence [34–36].

Cucumber (*Cucumis sativus* L.) is an important horticultural crop cultivated worldwide for its fresh and edible fleshy tissues [37]. As a result, its flavor plays a critical role in determining its commercial value. Despite this, the molecular mechanisms underlying astringency development in cucumber remain inadequately understood, and the role of *PAL* family genes in this process has not yet been reported. To date, research on cucumber *PAL* genes has focused mainly on stress responses [38]. In tea, astringency is mainly caused by tea polyphenols, and elevated expression of *PAL* in green tea increases polyphenols concentrations, leading to greater astringency [39]. To elucidate the role of *PAL* genes in cucumber astringency development, this study conducted a comprehensive genome-wide analysis of the *PAL* gene family in cucumber. This included gene structure characterization, protein secondary structure prediction, subcellular localization prediction, chromosomal mapping, *cis*-acting element identification, collinearity assessment, and evaluation of evolutionary selection pressures. In addition, transcriptional expression profiling of *PAL* genes was performed in cucumber fruits with varying levels of astringency, and the correlation between tannin content in cucumber fruits and *PAL* genes expression was examined. This study aims to advance the understanding of the molecular functions of *PAL* family genes in cucumber and to reveal their potential roles in the development of astringency in cucumber fruits.

## Materials and methods

### Plant materials

The cucumber specimens employed in this study were sourced from the greenhouse of the College of Horticultural Science & Technology at Hebei Normal University of Science & Technology, located at 119°10’ E, 39°42’ N. The experimental samples included strongly astringent cucumber varieties (‘21A114’, ‘21A127’, ‘HC’, ‘17S-135’, ‘17S-139’, ‘21A179’, ‘21A127-2’) and mildly astringent ones (‘17S-20’, ‘17S-23’, ‘17S-50’, ‘FC’, ‘LNTZT’), all of which belong to the South China-type cucumber. The astringency index (AI) of each cucumber material is shown in S1 Table [40]. Grading criteria for the cucumber astringency index (AI): AI = 0 indicates no astringency, AI < 40 indicates slight astringency, 40 ≤ AI < 70 indicates moderate astringency, and AI ≥ 70 indicates strong astringency. For each sample, fruit materials (sampled as a mixture of peel and pulp) were harvested 9 days post pollination and three biological replicates. These samples were promptly frozen in liquid nitrogen and temporarily stored at −80°C to facilitate subsequent experimental analyses.

### Genome-wide identification of the PAL gene family members

The PAL family members of Arabidopsis, tea, and cucumber were obtained from the online database TAIR (https://www.arabidopsis.org/), TPIA (https://tpia.teaplants.cn/) and CuGenDB (http://cucurbitgenomics.org), respectively. The Arabidopsis genome is from TAIR10.1, and its corresponding NCBI RefSeq assembly is GCF_000001735.4. The cucumber genome is from Cucumber_9930_V3, with the corresponding NCBI RefSeq assembly being GCF_000004075.3. The tea genome data is from the relevant information of Tieguanyin in the TPIA database. The HMMER software (version 3.4) was used for verification to obtain a gene set with high credibility. The final *PAL* gene set was obtained based on the conserved domains of PAL proteins.

### Gene structure and chromosomal localization

Based on the gene sequences of the *PAL* family, the structure of each *PAL* genes was obtained on the GSDS2.0 online software (https://gsds.gao-lab.org/index.php) [41]. Meanwhile, with the help of the results of local BLAST analysis, all *PAL* genes in each species were mapped to their respective chromosomal positions. Subsequently, each gene was systematically renamed to reflect its chromosomal location, so as to improve the clarity and organization of the genomic data for further analysis.

### Protein conserved domain and secondary structure analysis

We employed the online software MEME to analyze the conserved motifs within the PALs (https://meme-suite.org/meme/) [42]. The conserved motifs were visualized to highlight the conserved domains of the PAL family proteins using the online tool Evolview (https://www.evolgenius.info/evolview/#/) [43]. The online resource Expasy (https://web.expasy.org/) [44] was used to analysis of the secondary structure of these proteins.

### Phylogenetic analysis

The amino acid sequences of PALs were imported into the MEGA software (version 11.0.13) for sequence alignment using the ClustalW algorithm [45]. Subsequently, the Neighbor-Joining method was employed to construct a phylogenetic tree with a Bootstrap parameter set to 1000 replicates. The resulting newick (nwk) data were then analyzed further using the online software Evolview (https://www.evolgenius.info/evolview/#/), providing a visual representation of the evolutionary relationships among the *PAL* family members.

### *Cis*-acting element analysis

We developed a Python script (version 3.8.1) to extract 2000 base pairs upstream from each gene to obtain the promoter sequence. The *cis*-acting elements within these promoter regions were identified using the Plant Care online tool (https://bioinformatics.psb.ugent.be/webtools/plantcare/html/) [46] and visualized with the Evolview software (https://www.evolgenius.info/evolview/#/). The pheatmap package (version 1.0.12) in the R programming language (version 4.3.3) was employed to generate a heatmap analyzing the distribution and frequency of these *cis*-acting elements.

### Subcellular localization prediction

The amino acid sequences of all *PALs* were analyzed using WoLF PSORT (https://wolfpsort.hgc.jp/) [47] to predict their subcellular localization. The pheatmap package (version 1.0.12) in the R programming language (version 4.3.3) was utilized to create a heatmap.

### Collinearity analysis and Ka/Ks analysis

The collinearity analysis was carried out using software or tools such as jcvi software (version 1.4.16) [48], seqtk software (version 1.4), Bedtools (version 2.31.1) [49], Perl (version 5.22.0), and Circos (version 0.69.9) [50] respectively. For the Ka/Ks analysis, we first used the Simple Ka/Ks Calculator of TBtools (version 2.142) [51] to calculate the Ka, Ks, and Ka/Ks values for each gene pair. Based on these values, we employed the R programming language (version 4.3.3, with the plot3D package version 1.4.1 and RColorBrewer package version 1.1–3) to generate a three-dimensional scatter plot for further analysis.

### RNA isolation, RNA-seq, reverse transcription and qRT-PCR

In a previous report, we examined the astringency levels of fruits (9 days post-pollination, dpp) from 228 cucumber germplasm resources, ‘FC’ (low-astringency) and ‘HC’ (high-astringency) were screened [40]. To identify the major genes influencing cucumber astringency, we selected cucumber materials ‘HC’ and ‘FC’ for transcriptome sequencing. First, we use the RNAprep Pure Plant Kit (TIANGEN, China) to extract total RNA from cucumber fruits (9 days post-pollination), and assessed the extraction quality on a 1% agarose gel. Sample purity was determined using a NanoDrop ND2000 spectrophotometer. Select the total RNA from high-astringency cucumber material ‘HC’ and low-astringency cucumber material ‘FC’ for transcriptome sequencing. The total RNA was used to synthesize cDNA with the FastQuant RT reagent kit (Tiangen, China) for subsequent experiments. Then, to further verify the association between *PAL* genes and the formation of cucumber astringency, we selected 12 cucumber materials with extreme astringency traits (7 high-astringency cucumbers and 5 low-astringency cucumbers), and obtained the expression levels of *CsaPALs* in these materials through qRT-PCR. Real-time quantitative PCR was performed on a BIO-RED instrument using the SYBR^®^ Plus Universal qPCR Kit. The qRT-PCR program comprised an initial denaturation at 95°C for 2 minutes, followed by 40 cycles of 95°C for 5 seconds, 60°C for 10 seconds, and 72°C for 15 seconds (S2 Table). The *CsUBI* gene was selected as the internal reference gene, and relative expression levels were calculated using the 2^-ΔΔCt^ method. Statistical analysis (*p* < 0.05, one-way ANOVA, Tukey test) and graph generation were conducted using GraphPad Prism software.

### Determination of tannin content and correlation analysis

The tannins in cucumber fruits (sampled as a mixture of peel and pulp) were extracted with dimethylformamide (DMF) solution, followed by determination using a colorimetric method. Correlation analysis was carried out using SPSS software (Version 27.0.1.0).

## Results

### Genome-wide identification and gene structure analysis of *PAL*

In this study, a total of 23 *PAL* genes were identified (Table 1) across three species: *Arabidopsis thaliana* (4 genes), *Camellia sinensis* (6 genes), and *Cucumis sativus* L. (13 genes). The amino acid sequences encoded by these *PAL* genes ranged in length from 687 amino acids (*CsiPAL3*) to 725 amino acids (*AtPAL1*), with molecular masses varying between 74889.74 Da (*CsiPAL3*) and 78725.73 Da (*AtPAL1*). The physicochemical properties of these proteins were then analyzed, revealing that all PALs are acidic in nature (pI < 7). In addition, their GRAVY index values were all below zero, indicating that the proteins are highly hydrophilic. The analysis of chromosomal loci showed that *PAL* genes are predominantly clustered on cucumber chromosome 6, implying that potential tandem duplication events may have contributed to their expansion on this chromosome [52].

**Table 1:** Gene information of the *PALs* in various species.

Organism	Gene	Gene ID	Gene Locus	ORF(bp)	Amion Acid	Molecular Weight (Da)	pI	GRAVY	Position (Start-End)
*Arabidopsis thaliana*	*AtPAL1*	AT2G37040.1	Chr2	2178	725	78725.73	5.9	−0.156	15557367-15560755
	*AtPAL2*	AT3G53260.1	Chr3	2154	717	77859.93	6.03	−0.171	19744018-19747114
	*AtPAL3*	AT5G04230.2	Chr5	2097	698	76673.54	6.08	−0.155	1160569-1163957
	*AtPAL4*	AT3G10340.1	Chr3	2124	707	76919.61	5.88	−0.17	3203988-3208135
*Cucumis sativus* L.	*CsaPAL1*	CsaV3_1G040750	Chr4	2142	713	77979.19	6.13	−0.221	1392894-1395036
	*CsaPAL2*	CsaV3_4G002310	Chr6	2145	714	78033.18	6.37	−0.182	22800380-22803684
	*CsaPAL3*	CsaV3_4G002320	Chr6	2142	713	78054.03	6.15	−0.191	22819318-22821766
	*CsaPAL4*	CsaV3_6G014060	Chr6	2118	705	77172.26	5.96	−0.085	22831990-22834447
	*CsaPAL5*	CsaV3_6G036550	Chr6	2154	717	78235.39	6.18	−0.146	22824026-22826502
	*CsaPAL6*	CsaV3_6G039660	Chr1	2136	711	77874.09	5.51	−0.101	25961682-25964356
	*CsaPAL7*	CsaV3_6G039670	Chr6	2142	713	77640.70	6.23	−0.177	10244362-10248495
	*CsaPAL8*	CsaV3_6G039680	Chr6	2142	713	78373.23	6.26	−0.227	20347183-20350776
	*CsaPAL9*	CsaV3_6G039690	Chr4	2142	713	78067.89	6.00	−0.208	1387634-1390011
	*CsaPAL10*	CsaV3_6G039700	Chr6	2127	708	77546.46	5.76	−0.177	22815172-22817586
	*CsaPAL11*	CsaV3_6G039710	Chr4	2142	713	77941.73	5.93	−0.2	1382795-1385332
	*CsaPAL12*	CsaV3_6G039720	Chr6	2139	712	77793.65	5.80	−0.194	22810003-22812492
	*CsaPAL13*	CsaV3_4G002330	Chr6	2118	705	77236.40	6.12	−0.152	22827950-22830486
*Camellia sinensis*	*CsiPAL1*	GWHTASIV000402	Chr1	2130	709	77412.49	5.93	−0.145	21954637-21958295
	*CsiPAL2*	GWHTASIV014419	Chr4	2136	711	77399.4	6.21	−0.177	210978148-210981687
	*CsiPAL3*	GWHTASIV023860	Chr7	2064	687	74889.74	6.42	−0.153	195913658-195919863
	*CsiPAL4*	GWHTASIV032361	Chr10	2145	714	77724.8	6.01	−0.16	111281178-111286740
	*CsiPAL5*	GWHTASIV034458	Chr11	2112	703	76706.7	5.78	−0.126	60313456-60316189
	*CsiPAL6*	GWHTASIV039366	Chr13	2121	706	77078.23	6.26	−0.149	85477967-85483020

**Fig 1:**
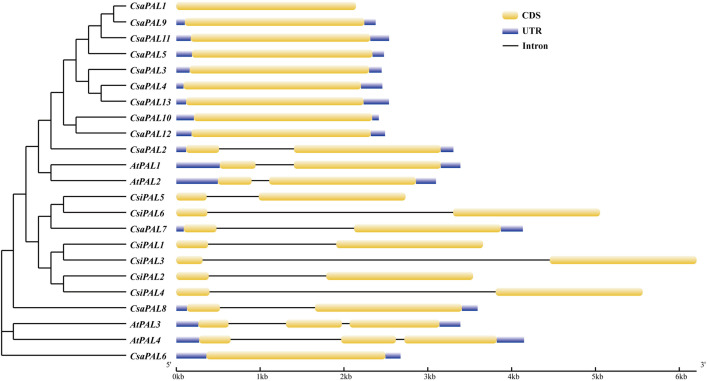
The gene structures of the *PAL*s. The yellow rectangles represent exons, the black solid lines represent introns, and the blue rectangles represent UTR.

To gain a deeper understanding of the *PALs*, gene structure diagrams were initially constructed. The results revealed that the gene lengths in the PAL family genes range from 2 kb to 6 kb. Specifically, in cucumber, the fragment sizes of *PAL* genes are predominantly between from 2 kb and 3 kb. In contrast, the tea gene *CsiPAL3* exceeds 6 kb, with introns comprising more than 50% of its length. The number of exons in *PAL* genes typically range from 1 to 3. In cucumber, most *PAL* genes contain a single exon, except for *CsaPAL2*, *CsaPAL7*, and *CsaPAL8,* which have two exons each (Fig 1).

### Analysis of conserved motifs and phylogenetic relationships of the PAL family proteins

**Fig 2:**
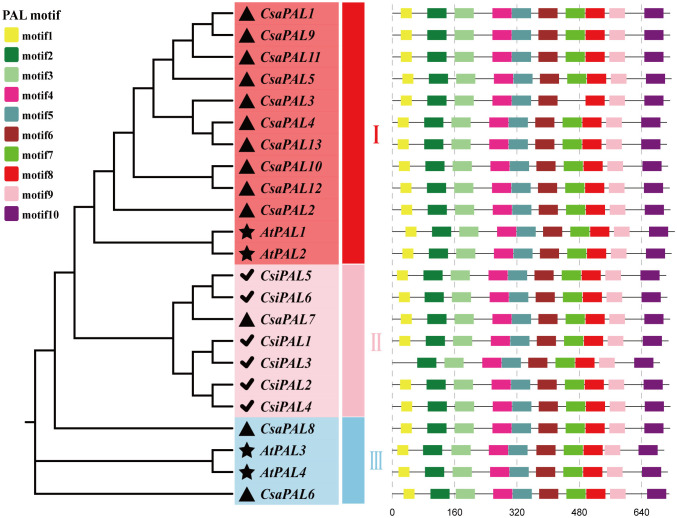
The composition of conserved motifs and phylogenetic tree of the PAL family proteins. The amino acid sequences of the PAL family are from Arabidopsis (4), tea (6), cucumber (13). The PALs of Arabidopsis were marked with black star, the tea is marked with a black check, and the cucumber is marked with a black triangle.

To explore the phylogenetic relationships among genes in the PAL families, the amino acid sequences of PAL proteins from Arabidopsis, tea, and cucumber were compared, and subsequently phylogenetic trees were constructed for each species (Fig 2). The analysis revealed that the 23 PALs clustered into three distinct groups, containing 12, 7, and 4 members, respectively. The evolutionary analysis indicated that 10 out of the 12 genes in group Ⅰ were derived from cucumber *PAL* genes (*CsaPAL1*-*CsaPAL5* and *CsaPAL9*-*CsaPAL13*), implying a close evolutionary relationship and high homology among them. In contrast, the 6 *PAL* genes from tea were clustered in group Ⅱ, showing a moderate degree of homology with the *CsaPAL7* gene in cucumber. Within the PAL gene families, 10 conserved protein motifs were identified (Fig 2, S3 Table). Motif 1 and 9 were 29 and 41 amino acids long, respectively, while all other motifs were 50 amino acids in length. Notably, *CsaPAL3* lacked motif 7, and *CsiPAL3* lacked motif 1. The presence of these conserved motifs across the PAL family proteins highlights the high degree of homology among *PAL* genes.

**Fig 3:**
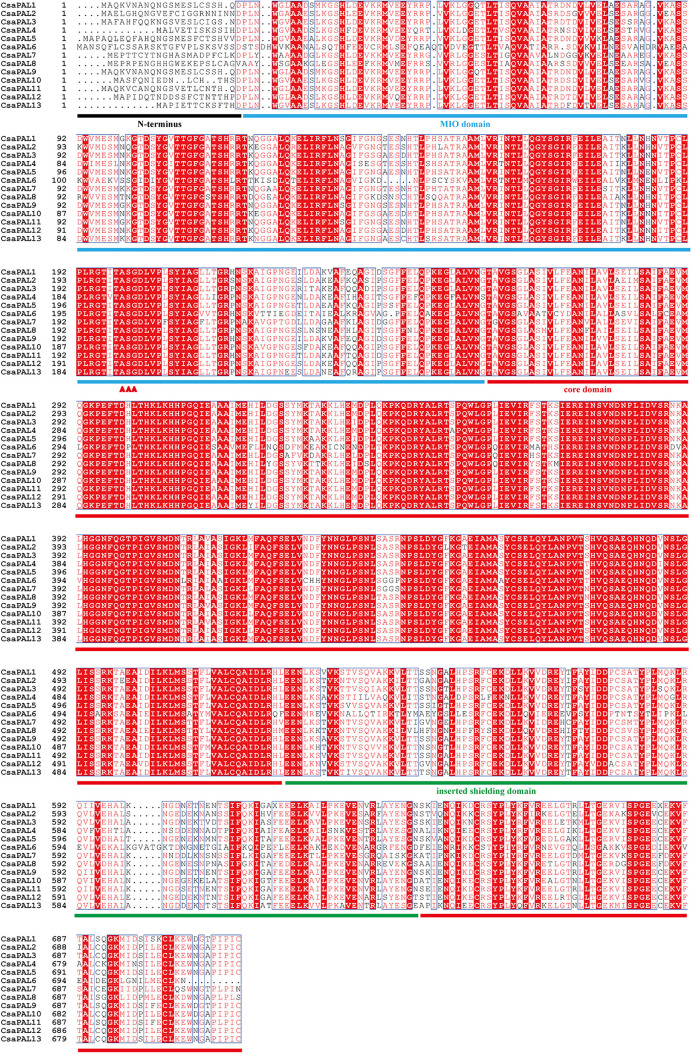
Multiple sequence alignment of cucumber PALs. The black solid line represents the N-terminus, the blue solid line represents the MIO domain, the red solid line represents the core domain, and the green solid line represents the inserted shielding domain. The conserved Ala-Ser-Gly (ASG) tripeptide motif specific to the PALs are marked with red triangles.

Previous studies have established that eukaryotic PAL proteins contain three conserved domains: the MIO domain, the core domain, and the inserted shielding domain. The MIO domain encompasses the MIO motif, which includes the catalytic triplet Ala-Ser-Gly [53–55]. To investigate cucumber PALs, we performed multiple sequence alignment of their amino acid sequences. All CsaPAL proteins were found to contain the N-terminal domain, the MIO domain, the core domain, and the inserted shielding domain, with the ASG tripeptide motif located in the MIO domain. These domain characteristics closely matched those previously reported for PAL proteins in plants (Fig 3).

### Analysis of the protein secondary structure and prediction of subcellular localization

The secondary structure of proteins plays a crucial role in determining their function. To better understand the potential functional characteristics of *PAL* genes, we analyzed the secondary structures of PAL family members (S4 Table), including α-helix, extended strand, β-turn, and random coil. The relative proportions of these structures varied among the PAL family members, with α-helix accounting for 50.19%−88.51%, extended strands for 4.91%−7.67%, β-turn for 1.15%−5.93%, and random coils for 10.34%−39.15%.

**Fig 4:**
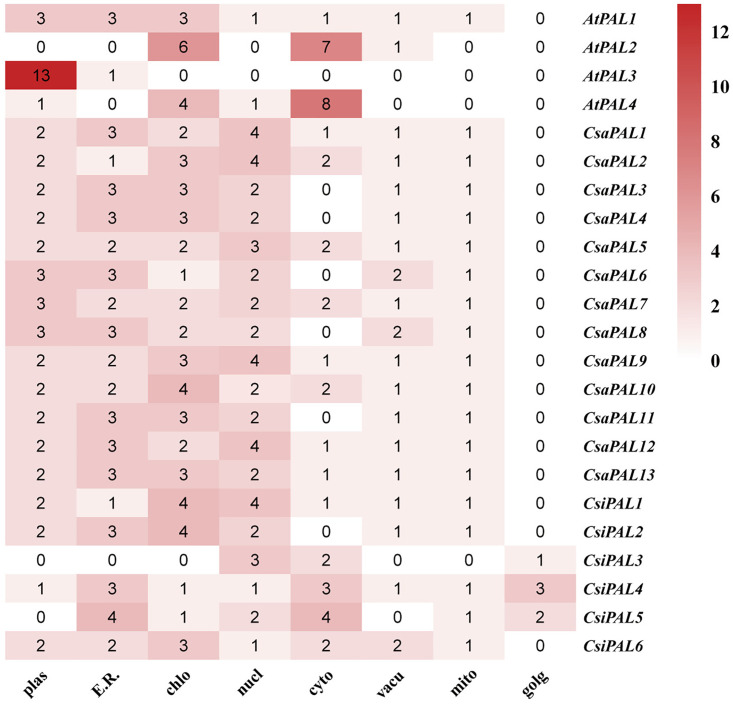
Subcellular localization prediction of the PAL family members. The larger the value, the higher the possibility that the protein is located in the organelle. ‘plas’ represents the plasma membrance, ‘E.R.’ represents the endoplasmic reticulum, ‘chlo’ represents the chloroplast, ‘nucl’ represents the nucleus, ‘cyto’ represents the cytosol, ‘vacu’ represents the vacuole, ‘mito’ represents the mitochondrion, ‘golg’ represents the golgi apparatus.

Predictions of subcellular localization indicate that these PALs are primarily found in eight cellular compartments (Fig 4); the plasma membrane, endoplasmic reticulum, chloroplast, nucleus, cytoplasm, vacuole, mitochondrion, and Golgi apparatus. In cucumber, CsaPAL proteins are predominantly localized in the plasma membrane, endoplasmic reticulum, chloroplast, and nucleus indicating that they may play important functional roles in these specific cellular locations.

### Chromosomal localization of the *PAL* genes

**Fig 5:**
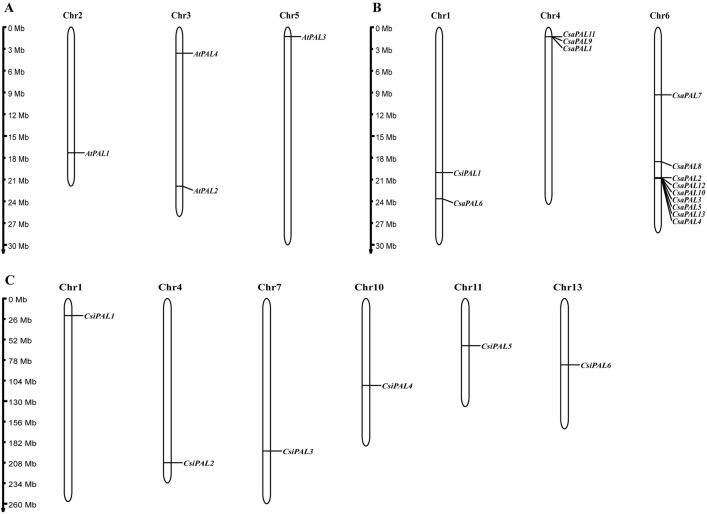
Visualized localization of the *PAL* genes on the chromosomes of each species. Chromosomal localization analysis of Arabidopsis **(A)**, cucumber **(B)**, and tea **(C)***.*

To visually examine the 23 *PAL* genes, their chromosomal locationswere mapped out. In Arabidopsis, the 4 *PAL* genes are distributed across chromosomes 2, 3, and 5 (Fig 5). In cucumber, the 13 *PAL* genes are located on chromosomes 1, 4, and 6, while in tea, the 6 *PAL* genes are positioned on chromosomes 1, 4, 7, 10, 11, and 13, respectively. Notably, in cucumber, *CsaPAL1*, *CsaPAL9*, and *CsaPAL11* form a cluster on chromosome 4, while *CsaPAL2*, *CsaPAL3*, *CsaPAL4*, *CsaPAL5*, *CsaPAL10*, *CsaPAL12*, and *CsaPAL13* cluster on chromosome 6. These two *PAL* gene clusters suggest high sequence similarity and potential functional conservation within the cucumber *PAL* gene family.

### Analysis of *Cis*-acting elements

**Fig 6:**
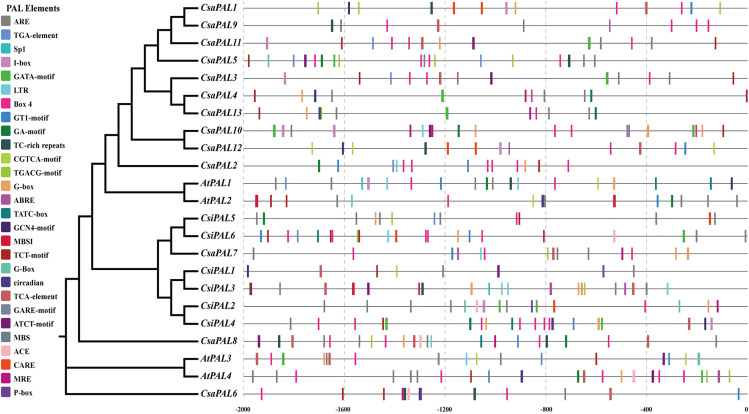
The distribution of *cis*-acting elements in the promoter regions of *PALs.* Different colors represent different *cis*-acting elements.

**Fig 7:**
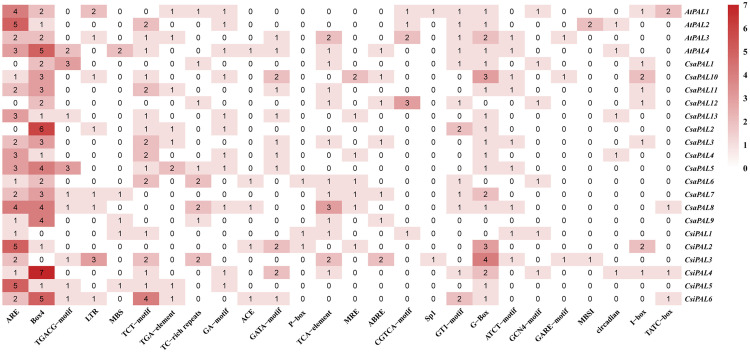
The distribution of the number of *cis*-acting elements in the promoter regions of the *PAL* genes.

The analysis of *cis*-acting elements within the promoter sequences (from −2000 bp to −1 bp) of *PAL* genes revealed 26 distinct *cis*-acting elements. These elements can be grouped into three main categories: light-responsive elements (Box4, G-Box, TCT-motif, GT1-motif, GATA-motif, MRE, I-box, ATCT-motif, GA-motif, Sp1, ACE), hormone-responsive elements (ABRE, TGACG-motif, CGTCA-motif, TCA-element, P-box, TGA-element, GARE-motif, TATC-box), and stress-responsive elements (ARE, LTR, TC-rich repeats, MBS, circadian). Of these, 24 elements were detected in the promoter sequences of cucumber *PAL* genes (Figs 6 and 7).

The *cis*-acting element analysis demonstrated that the Box4 element is present in the promoter regions of all cucumber *PAL* genes. Among them, *CsaPAL2*, *CsaPAL3*, *CsaPAL5*, *CsaPAL6*, *CsaPAL7*, *CsaPAL8*, *CsaPAL9*, *CsaPAL10*, and *CsaPAL11* showed relatively strong responses to the Box4 element. Additionally, *CsaPAL1* exhibited the most significant response to the TGACG-motif element, while *CsaPAL4* and *CsaPAL13* primarily responded to the ARE element (Fig 7). Overall, these findings suggest that members of the cucumber *PAL* gene family are involved in regulating responses to light, hormones, and various stresses.

### Analysis of gene duplication, collinearity and evolutionary selection pressure

**Fig 8:**
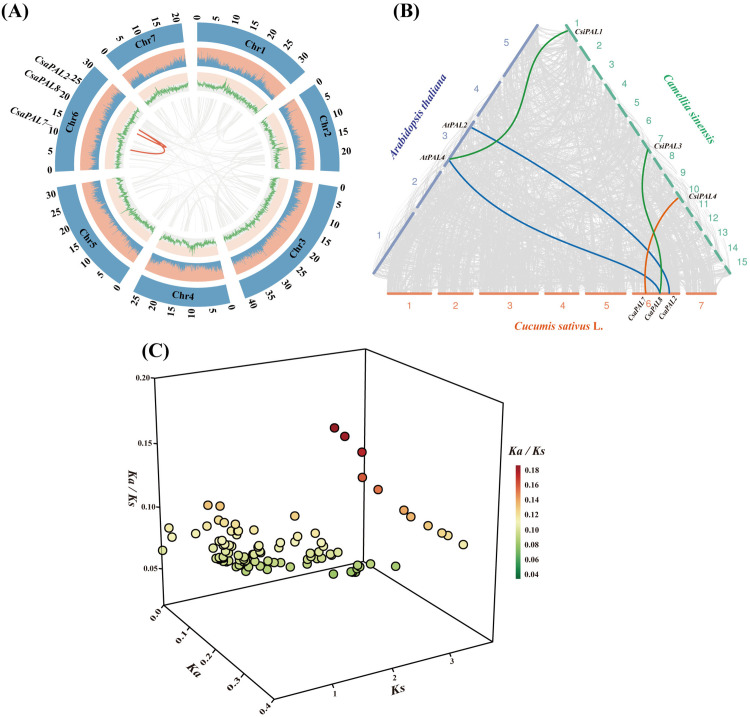
Analysis of gene duplication, collinearity and gene selection pressure of the *PAL* family. **(A)** Collinearity analysis within the cucumber genome. From the outside to the inside, they represent the distribution of each chromosome of cucumber, the distribution of the number of genes on each chromosome of cucumber, and the distribution of GC content in the cucumber genome. The gray and red arcs are the connections of blocks with collinearity. **(B)** Inter-species collinearity analysis of Arabidopsis, cucumber and tea. The gray, orange, green and blue arcs are the connections of blocks with collinearity. **(C)** Analysis of evolutionary selection pressure on homologous gene pairs of the *PAL* family. The x-axis, y-axis and z-axis represent the Ka value, Ks value and Ka/Ks value of the gene pairs respectively.

To explore the mechanisms of gene amplification within the *PAL* gene family, we conducted a collinearity analysis across the cucumber genome (Fig 8A). This analysis revealed that *CsaPAL2* has undergone tandem duplication events in conjunction with both *CsaPAL7* and *CsaPAL8*. This duplication could potentially influence the expression levels of the *PAL* gene. Furthermore, we performed a cross-species horizontal collinearity analysis of *PAL* family genes using cucumber, Arabidopsis, and tea. Collinearity blocks were constructed to visualize these relationships (Fig 8B). The results showed that *CsaPAL2* is collinear with *AtPAL2*, *CsaPAL8* is collinear with *AtPAL4* and *CsiPAL3*, and *CsaPAL7* is collinear with *CsiPAL4*. Additionally, *AtPAL4* exhibits collinearity with *CsiPAL1*. These relationships suggest that collinear genes may share similar or complementary functions across species.

To investigate the selection pressures acting on the *PAL* family genes during evolution, we calculated key evolutionary indicators for each gene pair: Ka (frequency of non-synonymous mutations), Ks (rate of synonymous mutations), and the Ka/Ks ratio. The Ka/Ks ratio serves as an indicator of selection pressures exerted on genes over time. The analysis revealed that all PAL gene pairs have Ka/Ks ratios below 1, implying that purifying selection has predominantly shaped their evolutionary trajectory.

### Expression profile analysis of *CsaPALs* genes and their correlation with tannin content

**Fig 9:**
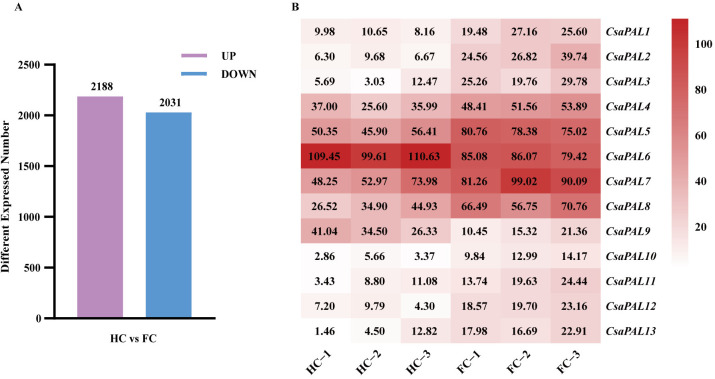
Analysis of differentially expressed genes in cucumber with different astringency levels. **(A)** The number of up-regulated and down-regulated DEGs of ‘HC’ vs ‘FC’. **(B)** Gene expression heatmap drawn based on the FPKM data from transcriptome sequencing results.

According to the RNA-Seq results, the comparison between ‘HC’ and ‘FC’ revealed 4219 differentially expressed genes (DEGs), including 2188 upregulated and 2031 downregulated genes (Fig 9A). Furthermore, the expression levels of 13 PAL family members showed significant variation between the two lines (Fig 9B). Specifically, *CsaPAL6* and *CsaPAL9* were expressed at significantly higher levels in ‘HC’ compared to ‘FC’, whereas the remaining *CsaPALs* genes displayed higher expression in ‘FC’ (Fig 9B).

**Fig 10:**
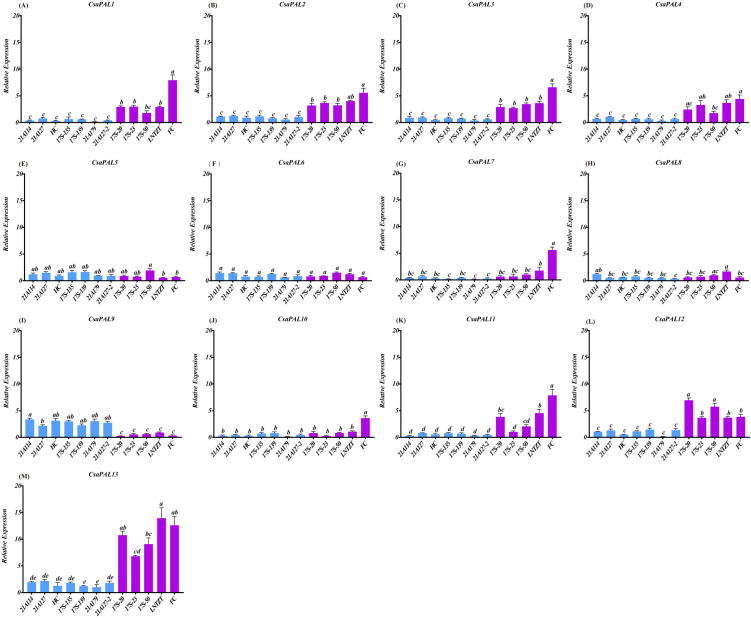
The expression profiles of the *CsaPALs* in cucumber with different astringency. Blue represents materials with high astringency, purple represents materials with low astringency. The cucumber *UBIQUITIN* gene was used as the reference transcript. Error bars represent the standard error from three independent experiments. Symbols a, b, c, d, and e indicate a significant difference at the *p*-value of 0.05.

**Fig 11:**
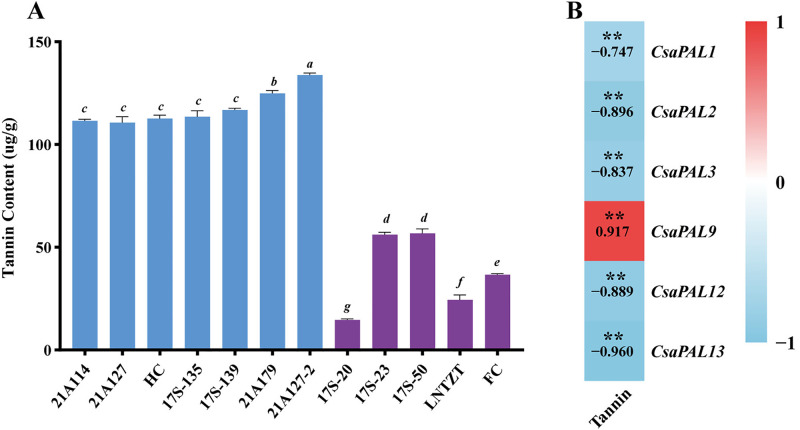
Correlation between tannin content and expression levels of *PAL* candidate genes in cucumber fruit materials with different astringency levels. **(A)** Tannin content in cucumber fruit materials with different astringency levels. Symbols a, b, c, d, e, f, and g indicate significant differences at the *p*-value of 0.05. **(B)** Correlation heatmap of tannin content and expression levels of *PAL* candidate genes. The values in the boxes represent Pearson correlation coefficients. ** indicates a significant correlation at the level of *p* < 0.01.

The qRT-PCR results showed that *CsaPAL1*, *CsaPAL2*, *CsaPAL3*, *CsaPAL12*, and *CsaPAL13* were expressed at significantly higher levels in low-astringency cucumbers compared to high-astringency ones. In contrast, *CsaPAL9* exhibited significantly higher expression in high-astringency cucumbers (Fig 10). These expression patterns aligned with the transcriptome sequencing results (Figs 9 and 10). Furthermore, high-astringency cucumbers generally contained more tannins than low-astringency ones, and the tannin content was significantly correlated with the expression levels of the six *PAL* genes (Fig 11). These findings suggest that *CsaPAL1*, *CsaPAL2*, *CsaPAL3*, *CsaPAL9*, *CsaPAL12*, and *CsaPAL13* are potential candidate genes involved in regulating cucumber astringency.

## Discussion

In the phenylpropanoid metabolic pathway, the enzyme PAL catalyzes the conversion of phenylalanine into cinnamic acid. As the initial enzyme in this metabolic pathway, PAL serves as a critical rate-limiting enzyme for the production of downstream metabolites and plays an essential role in tannin biosynthesis in plants [37]. Previous studies have extensively documented the genome-wide identification of *PAL* gene family members in various plant species [32,56–59]. In this study, we identified 13 *PAL* genes in cucumber (Fig 1). These genes exhibit structural variation, with exons counts ranging from 1 to 3 and variations in intron length and number (Fig 1, Table 1). This structural diversity may contribute to the functional differences, suggesting that different *CsaPAL* genes could participate in the synthesis of distinct secondary metabolites. For instance, in Arabidopsis, the *AtPAL1* and *AtPAL2* genes are mainly associated with flavonoid biosynthesis, while *AtPAL3* and *AtPAL4* are primarily involved in lignin biosynthesis [60]. Therefore, further research is required to clarify whether *CsaPAL* genes play a role in the biosynthesis of flavonoids and lignin in cucumber.

The plant *PAL* family genes are typically classified into three main sub-families [32,61,62]. In this study, we constructed a phylogenetic tree to illustrate the evolutionary relationships among PAL proteins from Arabidopsis, tea, and cucumber, and identified three distinct groups (Fig 2). The results showed that the 13 PAL proteins in cucumber are mainly distributed across two sub-families (Group I and Group III), a pattern consistent with findings in walnut [63], sorghum [64], and melon [65]. Furthermore, the *CsaPAL* genes in Group I share similar exon-intron structures and protein motifs, and cluster with *AtPAL1* and *AtPAL2*, which are known to function in flavonoid biosynthesis in Arabidopsis (Fig 2). This suggests that the *CsaPAL* genes in Group I may also play a role in flavonoid biosynthesis in cucumber.

The identification and analysis of *cis*-acting elements in gene promoter regions provides insight into gene transcriptional regulation and expression patterns. Research has demonstrated that the promoter regions of *PAL* family genes contain numerous *cis*-acting elements involved in hormone and stress responses [31,36]. In this study, the *cis*-acting elements in the promoter regions of *CsaPALs* genes were categorized into three main groups (Fig 6): light-responsive elements, hormone-responsive elements, and stress-responsive elements. Each *CsaPAL* gene promoter region contains at least one hormone response-related *cis*-acting element (Fig 7). Interestingly, while the ERE element is found in all *PAL* genes promoters in *Hevea brasiliensis* [64], it was absent in the promoter regions of *CsaPAL* genes, indicating that *cis*-acting elements in the promoters of the *PAL* family vary between species. Certain *cis*-acting elements appear exclusively in the promoter regions of specific *CsaPAL* genes (Fig 7). For instance, the P-box was detected in the promoter region of the *CsaPAL6* gene, the CGTCA-motif was found in the promoter region of *CsaPAL12*, and the GARE-motif was identified in the promoter region of *CsaPAL10* (Fig 7). Several studies have shown that light can influence the astringency of fruits. For example, during the growth of grapes, increasing light exposure and temperature through pruning can significantly enhance the astringency of grapes [53]. Conversely, reducing ultraviolet light exposure can lower the astringency of tea leaves [54]. Cherries grown outdoors exhibit stronger astringency compared to those grown in greenhouses [55]. High temperatures can intensify the astringency of fruits. Swede, a root vegetable, experiences an increase in fruit astringency at high temperatures, while the astringency is greatly reduced at low temperatures [66]. In addition, gibberellin and auxin also affect the astringency of tea leaves [67]. Therefore, the light-responsive, hormone-responsive, and stress-responsive *cis*-acting elements in the promoter regions of cucumber *PAL* genes may affect the expression level of the *PAL* gene, and thus influence the astringency of cucumbers. The exact functions of these *cis*-acting elements in the promoter regions of these genes still need to be further studied and clarified.

Analyzing genetic selection pressure plays an important role in clarifying the evolutionary processes of species. In this context, Ka represents the rate of non-synonymous mutations, referring to the frequency of mutations in a gene sequence that cause amino acid changes, while Ks represents the rate of synonymous mutation, reflecting the frequency of mutations that do not affect the encoded amino acid sequence [68]. The Ka/Ks ratio represents the relative frequency of these two mutation rates and is commonly used to assess the selection pressure a gene experiences during evolution. A Ka/Ks value less than 1 indicates that the main driving force for gene evolution comes from purifying selection, which removes deleterious mutations [68]. In this study, we analyzed the Ka/Ks ratios of *PAL* genes in Arabidopsis, tea, and cucumber. The results showed that the Ka/Ks ratios of all *CsaPALs* genes were less than 1 (Fig 8C), indicating that these *CsaPAL* genes are evolutionarily conserved, with stable structures and consistent functions [69]. Additionally, we conducted a genomic collinearity analysis of Arabidopsis, tea, and cucumber, which revealed collinear relationship between *AtPAL2* and *CsaPAL2*, as well as between *AtPAL4* and *CsaPAL8* (Fig 8B). Previous studies have demonstrated that the *AtPAL2* and *AtPAL4* genes of Arabidopsis are involved in the synthesis of flavonoids and lignin, respectively [60]. However, additional research is needed to determine whether *CsaPAL2* and *CsaPAL8* perform similar functions in the metabolism of flavonoid and lignin compounds in cucumber.

Catechins and flavonoids are the main substances that cause the astringency of tea [14]. During the maturation of tea leaves, the abundance of catechins is significantly positively correlated with the expression of *PALs* [70,71]. In albino tea plants (AnJiBaiCha), the expression of *PAL* is negatively correlated with the content of catechins [72]. Here, the expression levels of the 13 cucumber *PAL* genes in materials with different astringency levels showed that 6 *CsaPAL* genes might be related to the astringency of cucumber. Based on the expression patterns of *CsaPAL* in cucumber materials, the differential expression of *CsaPAL* genes between high- and low-astringency cucumber materials shows a “bidirectional regulation” pattern: the expression level of *CsaPAL9* in high-astringency materials (‘HC’) is significantly higher than that in low-astringency materials (‘FC’), while *CsaPAL1*, *CsaPAL2*, *CsaPAL3*, *CsaPAL12*, and *CsaPAL13* show the opposite trend (higher expression in low-astringency materials) (Fig 10). Additionally, the expression levels of these six genes are significantly correlated with the fruit tannin content (Fig 11B). These results suggest that there may be a “functional division” in the regulation of astringency within the cucumber *PAL* gene family: *CsaPAL9* may contribute to the high-astringency phenotype by positively regulating tannin synthesis, while the other five genes may indirectly affect the astringency level by negatively regulating tannin accumulation or diverting the metabolic flux towards other phenylpropanoid derivatives (such as lignin). Similar functional differentiations have been reported in other plants. For example, in Arabidopsis, *AtPAL1* and *AtPAL2* are mainly involved in the synthesis of flavonoids, while *AtPAL3* and *AtPAL4* are more involved in lignin synthesis [60]. This functional division may be an adaptive strategy developed by the *PAL* gene family through gene duplication and functional diversification during long-term evolution to meet the diverse demands for phenylpropanoid metabolites in different tissues and at different developmental stages. It is worth noting that the seven *CsaPAL* genes (*CsaPAL4*, *CsaPAL5*, *CsaPAL6*, *CsaPAL7*, *CsaPAL8*, *CsaPAL10*, *CsaPAL11*) with no significant expression differences detected in this study are not necessarily non-functional. They may play roles in other developmental stages or specific tissues of cucumber. For example, *CsaPAL8* shows collinearity with *AtPAL4* in Arabidopsis, which is mainly involved in lignin synthesis during stress responses (Fig 8B). It is speculated that when cucumbers face adverse conditions (such as low temperature, diseases), *CsaPAL8* may be highly expressed to promote lignin accumulation and improve plant resistance, rather than regulating fruit astringency. The specific functions of these genes in the formation of cucumber astringency still need to be further verified.

Compared with previous studies on the phenylalanine ammonia-lyase (PAL) gene family [38], we combined the cucumber-specific online database CuGenDB with the hidden Markov model of the PAL domain (PF00221.24) to identify 13 cucumber *PAL* genes (*CsaPAL1-CsaPAL13*), covering the cucumber *PAL* gene family more comprehensively. Additionally, the study by Amjad et al. focused on the regulatory role of *PAL* in cucumber thermotolerance, while our study emphasizes the regulation of cucumber astringency quality by *PAL* family genes and has identified candidate genes. The results of this study not only provide new perspectives for a deeper understanding of the molecular mechanism of cucumber astringency formation but also offer target genes for further improving cucumber astringency traits through methods such as gene editing and marker-assisted breeding.

## Conclusions

In this study, we identified 13 members of the *PAL* gene family in cucumber and investigated their physicochemical properties, protein structures, conserved motifs and domains, *cis*-acting elements, phylogenetic relationships, evolutionary selection pressures, collinearity, and expression patterns, and correlation analysis between the tannin content and the expression levels of *PAL* genes. Our findings demonstrated that cucumber *PAL* genes are highly conserved and contain specific conserved domains characteristic of eukaryotic plant *PAL* genes. Transcriptome sequencing analysis revealed substantial differences in the FPKM expression levels of 13 *PAL* genes between the high-astringency cucumber ‘HC’ and the low-astringency cucumber ‘FC’. Furthermore, qRT-PCR verification confirmed that six genes, *CsaPAL1*, *CsaPAL2*, *CsaPAL3*, *CsaPAL9*, *CsaPAL12*, and *CsaPAL13,* were significantly differentially expressed between ‘HC’ and ‘FC’. Additionally, the expression levels of these six genes were significantly correlated with cucumber fruit tannin content. These results suggest that these six genes are likely the candidates underlying cucumber astringency. This study laid a foundation for further exploration of the biological functions of cucumber *PAL* genes and provided valuable references for cucumber breeding aiming at astringency traits.

## Supporting information

S1 Table Evaluation information of astringency index for the experimental materials of cucumber. (XLSX)

S2 Table The primers used in qRT-PCR. (XLSX)

S3 Table The detailed information of the conserved motifs of the PAL family proteins. (XLSX)

S4 Table Detailed information on the secondary structure of the PAL family proteins. (XLSX)
